# Sensor Topology Optimization in Dense IoT Environments by Applying Neural Network Configuration

**DOI:** 10.3390/s23125422

**Published:** 2023-06-08

**Authors:** George Papastergiou, Apostolos Xenakis, Costas Chaikalis, Dimitrios Kosmanos, Periklis Chatzimisios, Nicholas S. Samaras

**Affiliations:** 1 Department of Digital Systems, University of Thessaly, 41500 Larissa, Greece; kchaikalis@uth.gr (C.C.); dikosman@uth.gr (D.K.); nsamaras@uth.gr (N.S.S.); 2 Department of Electrical & Computer Engineering, University of Thessaly, 38221 Volos, Greece; 3 Department of Information and Electronic Systems Engineering, International Hellenic University, 57001 Thessaloniki, Greece; pchatzimisios@ihu.gr

**Keywords:** sensor placement, topology, WSNs, network lifetime, neural networks

## Abstract

In dense IoT deployments of wireless sensor networks (WSNs), sensor placement, coverage, connectivity, and energy constraints determine the overall network lifetime. In large-size WSNs, it is difficult to maintain a trade-off among these conflicting constraints and, thus, scaling is difficult. In the related research literature, various solutions are proposed that attempt to address near-optimal behavior in polynomial time, the majority of which relies on heuristics. In this paper, we formulate a topology control and lifetime extension problem regarding sensor placement, under coverage and energy constraints, and solve it by applying and testing several neural network configurations. To do so, the neural network dynamically proposes and handles sensor placement coordinates in a 2D plane, having the ultimate goal to extend network lifetime. Simulation results show that our proposed algorithm improves network lifetime, while maintaining communication and energy constraints, for medium- and large-scale deployments.

## 1. Introduction

Sensor node deployment is a key design issue related to planning a wireless sensor network (WSN) and is closely related to the domain application requirements and the total energy consumption and robustness of the network. An optimal deployment may reduce communication costs and extend the total network lifetime, under certain constraints imposed by the networking elements and the application. In this way, a WSN can better fulfil its operational tasks, such as sensing and evaluating physical phenomena, data transmission, and inference [1].

Sensor deployment methods are closely related to specific applications [2] and can be of two types: deterministic and random. In the first type, the nodes’ coordinates are determined a priori by the network design team; this type is preferred in cases where the environment is not harsh. For example, in a precision agriculture application, the sensors’ locations may coincide with trees’ locations [3]. On the other hand, in cases where the physical phenomenon is mobile, the environment is harsh, or there are quick alterations in the sensing values, a random deployment is proposed [1,2]. To achieve the desired coverage ratio, redundant nodes are present, which means that the design is resistant to several node failures. For example, a random deployment is better in cases of measuring chemical gases inside a volcano, when estimating sudden weather changes, or when a cultivation is thick [3,4]. In the case of random deployments, the coverage constraint requirements may be not so strict. Comparing the two deployment types, one can say that the deterministic case needs careful planning, time, and resources. Moreover, the size of the set holding all possible network topologies is an exponential function of the size of the used sensor nodes. Therefore, deterministic planning is not always cost-effective. On the other hand, in random deployments, there is a high risk of network outage or partition in cases of total ad hoc deployments [5].

In this work, we apply the Voronoi diagram partitioning theory, as in [4], which is a deterministic approach and is geometrically designed to cover a certain terrain. Following this, we compare it via simulations and propose use case scenarios with random topologies (i.e., via Gaussian coordinate distribution), as in [6], and we give our results in terms of the overall network lifetime, communication, and coverage constraints. Our proposed optimization solver consists of several neural network configurations, with which we experiment so as to reach a near-optimal solution in polynomial time.

For our WSN communication model, we apply a realistic power consumption model of a wireless communication subsystem, which has been proposed in many related works [7,8,9]. The model introduces the energy consumption for sending and receiving a *P*-bit packet over a distance *d*. The energy required in wireless communication is related to the path loss model and is proportional to a loss factor of *d*^2^ for free space and *d^a^* for multipath fading and obstacles [7]. According to the use case scenario, the terrain space, and the node volume, there are two dominant communication patterns: in the first one, all nodes communicate directly with the gateway in an one-hop fashion, and in the second one, a multihop scenario is applied, especially to keep the communication range low and, thus, the energy consumption low. However, in this case, we need many relay nodes and this may lead to a lower total network lifetime [8]. Thus, the network lifetime is closely related to communication energy consumption, as in the majority of WSN deployment cases, nodes have limited energy resources. Following on from this, radio communication to guarantee a connected network data flow graph is mainly affected by the transmission distance and the transmission power. Thus, sensor topology optimization is closely related to the optimization of both the sensor deployment strategy and the applied communication range [9].

The research community shows a strong interest in applying AI tools and neural network (NN) methods to ensure energy efficient WSN deployments [10]. In essence, NNs can play a dominant role in this direction due to their quite simple and parallel-distributed computation design, robustness, and autoclassification methods in the case of sensor data collection. Dimensionality reduction, sensor data classification, and behavior prediction may lead to lower communication costs and overall energy conservation. In this paper, we define a joint sensor network energy and coverage constraint lifetime optimization problem and approximate its near-optimal solution in polynomial time, applying NN configurations as a solver. To this end, we consider and compare several topologies, in conjunction with NN configurations, by relocating certain sensor nodes to new coordinates, thus satisfying the communication and coverage constraints. We evaluate our solution by testing several use case scenarios, highlighting the impact of WSN escalation (i.e., small, medium, and large network deployments) and communication patterns on the overall network lifetime. The simulation results show that our proposed algorithm improves the network lifetime while maintaining communication and energy constraints for small-, medium-, and large-scale deployments. Finally, our solver proposes a final topology deployment in each use case.

The structure of the paper is as follows: In Section 1, we give an Introduction related to the problem under investigation. In Section 2, we discuss related research works and motivation. In Section 3, we discuss the details about the system model formulation, the neural network configuration, and the optimization framework. In Section 4, we give details about the proposed use cases and we present simulation parameters and results. Finally, in Section 5, we conclude this work and provide insights about future extensions.

## 2. Related Works and Motivation

A WSN designer places sensor nodes inside a field of interest (FoI) according to the application coverage requirements. These nodes, due to their transceivers, have the ability to cover either small or medium areas by sending data to a gateway node. The energy required for data communication is usually greater than the circuitry energy consumption [11]. In the majority of WSN and IoT applications, nodes are battery-equipped [6]. However, in cases where there is great difficulty in replacing batteries (i.e., harsh terrain, a large volume of nodes, etc.), researchers’ interest focuses on mechanisms to extend the network’s lifetime under communication and coverage constraints. Topology control plays a dominant role in achieving this target. Thus, optimal sensor node placement within the FoI is needed for cost-effective deployment.

The authors in [12] propose a sensor placement algorithm, which utilizes a biologically inspired optimization technique to imitate the behavior of territorial predators in marking their territories with their odors, known as the territorial predator scent marking algorithm (TPSMA). The TPSMA technique is based on maximizing a coverage objective optimization function. The problem of determining the location of sensor nodes, such that the terrain target points are covered and the network lifetime is maximized, is called the sensor deployment problem (SDP). Various heuristics and approximation algorithms have been proposed to solve the SDP in WSNs. Two improved versions of the particle swarm optimization (PSO) algorithm are presented in [13]. The first one is a cooperative PSO and the second is its improved version, applying fuzzy logic. These approaches do not deal with maximizing the coverage area or prolonging the network lifetime.

A properly designed and applied sensor deployment strategy improves WSN performance and resource management [9]. The coverage ratio is directly influenced by the deployment strategy. In principle, there is no positive correlation between energy consumption minimization and coverage maximization. Thus, to maximize the coverage area, sensor nodes should be placed far away from the sink node (SN) or the gateway node, which takes higher transmission power to reach the SN and raises the total energy consumption. This problem is partially solved if an energy-efficient and multihop routing algorithm is applied [7] instead of a one-hop communication pattern. However, in several precision agriculture applications [3], the terrain’s structure may block multihop communication patterns (i.e., tall trees) and, thus, the one-hop pattern along with an energy- efficient topology design may solve the energy problem. In essence, in this work, we follow this design pattern.

Following on from this, nature-inspired optimization algorithms are adopted by many researchers in WSN applications [14]. While genetics, ants, and particle swarm algorithms are the dominant examples, many others emerge regularly such as the flower pollination algorithm (FPA), which is a novel global optimization algorithm inspired by the pollination process of flowers [14]. Based on the multiobjective version of the FPA (i.e., MOFPA) for WSNs, a new approach is proposed in [15]. This approach aims to find the optimal topology deployment, taking into consideration conflicting objectives, such as total energy minimization and total coverage maximization, while maintaining connectivity constraints.

The network’s lifetime primarily depends on the total energy consumption, which is mainly related to the nodes’ radio electronics energy consumption. An energy-efficient coverage optimization technique, the Voronoi–glowworm smarm optimization–K-means algorithm, is presented in [16]. In this approach, the Voronoi cell structure enhances the area coverage, applying the minimum required number of active nodes. The Voronoi diagram is one of the most famous computational geometrical structures applied in sensor topology design problems to ensure coverage extension [17]. Following on from this, a sensor node deployment technique is proposed in [18] as a constrained multiobjective optimization (MOO) problem. The proposed algorithm is a multiobjective evolutionary algorithm (MOEA), known as MOEA/D-DE, that uses a decomposition approach and employs differential evolution (DE). The aim is to find a sensor node deployment to maximize the coverage rate, minimize the network energy consumption, maximize the network lifetime, and minimize the number of deployed sensor nodes, while ensuring connectivity between each sensor node and the sink node for proper data transmission. A tree structure between the deployed nodes and the sink node for data transmission is assumed in this approach.

Heuristic approaches are appropriate for near-optimal solutions, especially in NP-complete problems. To this end, a metaheuristic algorithm is proposed in [19], which determines the sensors’ positions for area coverage maximization. As a part of the solution, the authors utilized the immune plasma algorithm (IPA), a unique metaheuristic algorithm that is based on the immune plasma therapy concept and the transfer of an antibody-rich fraction of blood from previously recovered patients to others who are considered critically ill. This optimization algorithm is used to determine the optimal solution to the maximum coverage problem, but does not deal with the minimum number of nodes, network lifetime extension, or connectivity assurance.

The authors in [20] propose an approach which relies on exploiting the building information modeling (BIM) database to obtain real-time and valid information about a target area. The majority of the proposed schemes present possible solutions without taking into account the terrain structure and potential obstacles during the WSN topology design process. The proposed solution can be integrated as a plugin within BIM tools in order to optimize sensor deployment in real time, taking into account both nodes and obstacles, respectively. In order to optimize WSN deployment, after collecting useful data from sensor nodes and the BIM database, this approach relies on an evolutionary algorithm to solve the multiobjective problem for coverage, cost, and lifetime. The output is each sensor’s optimal location in the smart building application.

One of the major challenges in sensor deployment is to find the trade-off between conflicting network optimization objectives under certain connectivity constraints. As proposed in [21], an approach to deal with this is a constrained Pareto-based multiobjective evolutionary approach (CPMEA). It aims to find Pareto-optimal layouts which maximize the coverage and minimize sensors’ energy consumption to prolong the network lifetime, while maintaining full connectivity between each sensor node and the gateway. To cover any type of FoI with a predefined number of sensors, a genetic algorithm is proposed in [22] with the purpose of finding the best sensor placement while ensuring maximum network coverage under sensor connectivity constraints. The authors propose the genetic algorithm for area coverage maximization (GAFACM), which covers all shapes of areas for a given number of sensors and finds the best positions to maximize coverage within the FoI, while ensuring connectivity between the sensor nodes.

An additional WSN deployment approach, based on the gradient method and the simulated annealing (SA) heuristic algorithm, is proposed in [23], using the minimum number of sensor nodes. However, this work does not deal with maximizing the network lifetime, whereas in [24], the SA heuristic algorithm is applied along with an energy-efficient algorithm to arrange the placement of sensors in order to extend the network lifetime. The main function of WSNs is to gather the required information, process it, and send it to remote gateways. A large number of sensor nodes need to be deployed within a field of interest; therefore, finding the best node placement according to several constraints is a hard problem to solve because it escalates. Recent studies in [25] focus on solving the deployment problem by applying heuristic and metaheuristic optimization algorithms. In approximately 35% of these studies, the authors apply an improved version of swarm optimization algorithms to solve the sensor deployment problem under constraints. However, network scalability and total energy consumption are not always addressed.

The topology optimization problem in large IoT and WSN deployments is a combinatorial and NP-hard problem to solve in polynomial time. The majority of existing algorithms apply heuristic or nature-inspired rules to reduce the search number of potential problem solutions within a solution space, so as to obtain a suboptimal solution in polynomial time. Recently, researchers have started investigating the application of neural network methods to solve the sensor deployment problem (SDP). Therefore, to the best of our knowledge, these works are mostly a work in progress. In [26], the authors propose a deep-reinforcement-learning-based topology optimization problem for energy-efficient and self-organizing WSNs. According to simulations, they show that the proposed algorithm achieves better performance as compared to other heuristic solutions. Furthermore, the energy-efficient topology control problem in large WSNs is investigated in [27], with a noncooperative game theoretic approach. The extensive simulation results verify the validity of a utility function, which effectively balances the transmit power, residual energy, and network connectivity.

In our work, we follow the direction of investigating and applying several neural network configurations as a solver for our proposed optimization problem under constraints. The difference from [26] is related to the type of neural network solver and the network optimization problem with constraints and scalable network deployments. Compared with [25], this work focuses on solving a certain WSN deployment problem with energy and communication constraints. The proposed solution is based on several neural network configurations and not on a heuristic algorithm. In the following section, we provide details about our system model and optimization framework. We also discuss our neural network solver parameters, which propose which sensor nodes need to be relocated so that the objective function stays within acceptable limits.

## 3. System Model Design and Optimization Framework

### 3.1. System Model

We consider a field of interest (FoI), which is a 2D terrain, with an area of M × L m^2^. Inside the terrain, we place *N*, $N\;\in \;Z^{+}$ in a total wireless sensor network and they form a connected WSN, with one sink node *H*. The sink node collects all data and is placed at the centroid of the terrain. The nodes are considered homogeneous in terms memory, processing, communicating capabilities, and energy reserves. Their job is to periodically collect data samples and send them to the sink node (i.e., the base station—BS). Initially, the WSN is considered static, which means that no mobility is present, nor do the nodes change their coordinates. Additionally, each node has the same sensing range, $R_{s}$, and communication range, $R_{c}$.

**Figure 1:**
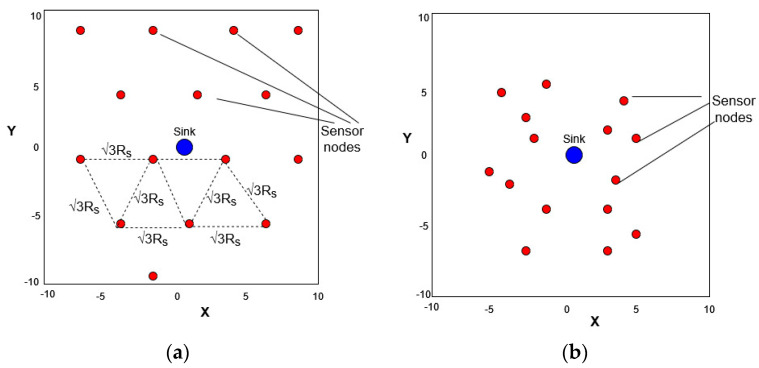
(**a**) Initial placement of sensor nodes for a deterministic deployment, sink node at point (0, 0); (**b**) Initial placement of sensor nodes for a random deployment, sink node at point (0, 0).

In Figure 1, we present an example of two topologies for a small-scale network case, the size of which is 20 × 20 m^2^, in which fifteen nodes are distributed. Let us call topology (a) the deterministic deployment of nodes, as produced by the centroidal Voronoi tessellation (CVT) [1], and topology (b) the random deployment, in which the nodes’ coordinates are derived by a Gaussian distribution [2]. The position of each node is stored in a discrete set of (x,y) coordinates. Therefore, the WSN is considered as a graph G(V,E), where V expresses the number of nodes, *N*, and E is the total edges that connect the nodes. Our network system model has the following design features:

1. All sensors are responsible for monitoring an event inside their sensing area, periodically taking measurements, and sending them to the BS. When they perform that, they are considered active.
2. Topologies in which a node has no neighbor are neither considered connected nor valid. Each sensor $i\in \left[1\ldots N\right]$ should communicate with at least one neighbor and send the data to the BS, either directly or via a multihop pattern [5]. In the case of a multihop pattern, relay nodes send all packets to the BS.
3. The coordinates (x,y) of each sensor may change over time due to the neural network solver decision to minimize the total energy consumption.
4. The position of the BS is stationary.
5. We call the initial sensor positions theoretical points, which constitute the CVT topology, in which the optimal sensor distance is $d_{uv}\leq \sqrt{3}R_{s}$ [1]. We also call each sensor’s coordinate query points (i.e., application reserved coordinates) as the application domain dictates and as long as there is a WSN-connected graph at all times. For example, in a smart agriculture application, we may want to mount each sensor at certain terrain locations, i.e., on a tree.
6. The area to be monitored is a rectangular 2D grid terrain of size M×M, where $M\in Z^{+}$.
7. We define a round as a case where all sensor nodes in the WSN send at least one packet towards the gateway node. Additionally, we define the network lifetime as the total rounds until all nodes reach zero energy reserve (i.e., all the nodes die).

### 3.2. Problem Formulation

In this work, we focus on maximizing the 2D sensing area by activating the minimum number of sensors and selecting their optimal coordinates in terms of extending the network lifetime under energy and communication constraints. We, therefore, propose a joint optimization problem to minimize the total energy consumption and maximize the coverage area by ensuring a WSN-connected graph at all times. All possible generated topologies generated randomly are a function of *N*, as $\left(\begin{array}{c} M\times M\\ N \end{array}\right)$. Therefore, the number of topologies scale exponentially as a function of the number of scattered nodes. However, only a subset of the produced topologies are valid ones, thus satisfying the network constraints. To do so, the overall network energy consumption must be minimized and, thus, the total residual energy is maximized and the WSN’s lifetime is extended.

In our model, we apply the first-order radio model [27] in which the energy of the radio transmitter and receiver circuitry is equal to $e_{elect}$ = 50 nJ/bit for the electronic subsystem, and $e_{amp}=\frac{100\mathrm{pJ}}{\mathrm{bit}}$ $m^{2}$ expresses the energy required to run the transmitter amplifier. The model also assumes a pass loss factor $\approx \frac{1}{d^{l}}$, where *d* is the Euclidean distance between two nodes and *l* is the path loss exponent as 2≤l≤5, according to the application’s requirements. The energy consumed to transmit a packet is given as:

(1) $$E_{tx}=e_{elect}\times k+e_{amp}\times k\times d^{l}$$

where *k* is the packet size in bits. Following this, the energy consumed to receive a packet of *k* bits from a node is given as:

(2) $$E_{rx}=e_{elect}\times k$$

According to Equations (1) and (2), the total energy consumption per node is defined as: $E_{total}=pk*\left(E_{tx}+E_{rx}\right)$, where *pk* stands for the total number of packets sent or relayed by a sensor node. Additionally, we assume that the radio channel is symmetric, which means that the energy cost of transmitting and receiving a packet is the same. As far as the path loss channel model is concerned, we assume the log-normal shadowing model, as in [28]. Empirical studies [28] have shown that the log-normal shadowing model provides a more accurate multipath channel model as compared to Nakagami and Rayleigh, appropriate for a cellular deployment. Therefore, the path loss is given as:

(3) $$PL\left(d_{ij}\right)=PL\left(d_{o}\right)+10\beta \;\log_{10}\left(\frac{d_{ij}}{d_{o}}\right)+X_{\sigma }$$

where $d_{o}$ is a reference distance, *β* is the path loss exponent, and $X_{\sigma }$ is a zero-mean Gaussian random variable in dB, with a standard deviation, *σ*, to simulate the shadowing effects. Following this, for each node given a transmitting power, $P_{t}$, in dBm, the received power in dBm is given as follows:

(4) $$P_{r}=P_{t}-PL\left(d_{ij}\right)$$

We formulate a joint energy and coverage optimization problem, with connectivity constraints, in which the objective function, $f_{1}$, relates to the total average WSN energy consumption for the data collection and transfer to the BS and $f_{2}$ relates to the total sensing coverage inside the given terrain. The definition of $f_{1}$ is:

(5) $$f_{1}=\frac{1}{N}\sum_{i\in S}E_{total}$$

where the set *S* defines the set of all active nodes. The sensor connectivity constraint is fulfilled, as long as $P_{ri}\geq P_{rthres}\;\forall i\in \left[1\ldots N\right]$ and $P_{rthres}$ gives the minimum required power for the packet reception. Furthermore, the second objective relates to the maximization of the coverage area, in essence the maximization of the total sensing coverage points within the terrain. To do so, we apply the binary sensing model [29,30,31] and consider the following function to measure the overall overage:

(6) $${NC}_{ovp}=\left\{\begin{array}{l} 1,\;d\left(S_{i},m_{p}\right)>R_{s}\\ 0,\;otherwise \end{array}\right.$$

where $R_{s}$ is the sensing range and *d*(*s_i_*,*m_p_*) is the Euclidean distance between the monitoring point ($x_{mp}$, $y_{mp}$) and the sensor node *i*’s coordinates ($x_{si},y_{si})$. The coverage constraint is fullfilled as long as, for each produced topology *j*, we minimize the Euclidean distance between the monitoring point and the sensor’s coordinates, i.e., *min*$d\left(s_{i},m_{p}\right)=\sqrt{{\left(x_{si}-x_{mp}\right)}^{2}+{\left(y_{si}-y_{mp}\right)}^{2}}$. We define the objective function, $f_{2}$, as follows:

(7) $$f_{2}=\sum_{p\in M}NC_{ovp}$$

where *M* is the total number of monitoring points. Moreover, there is a constraint within our optimization problem. It relates to the fact that the distance between any two sensor nodes should not exceed their communication range, *R_C_*. Additionally, only one sensor node is placed in each monitoring location. We name each monitoring location *x*(*p*) to indicate whether the location is equipped with a sensor or not, as in [7]. Following a binary representation, *x*(*p*) is defined as:

(8) $$x\left(p\right)=\left\{\begin{array}{l} 1,\;\mathrm{if}\;\mathrm{location}\;p\;\mathrm{has}\;a\;\mathrm{sensor}\;\mathrm{node}\\ 0,\;\;\;\mathrm{otherwise} \end{array}\right.$$

In the case of deterministic deployment, applying the centroidal Voronoi tessellation (CVT), nodes are scattered and form an optimal geometry deployment, as near as possible to the gateway, which subsequently means fewer transmission costs for the entire network [8]. To ensure the proper functionality of the deployed WSN, we make the following assumptions:

1. To ensure connectivity, the communication radius, *R_c_*, is at least two times the sensing radius, *R_s_,* which is *R_c_* ≥ 2 × *R_s_.* In this condition, we only need to consider the coverage problem in the sensor network; if the network is covered, then it is connected [9].
2. Given that the nodes with a sensing radius, *R_s_*, are deployed in the triangular mesh configuration in the sensing field, *A*, the coverage fraction, *k*, of the sensing field varies nonlinearly with the spacing between two adjacent nodes, in the range $\sqrt{3}$*R_S_* ≤ *d* ≤ *2R_S_*, where *d* is the internode distance [10].
3. For a given coverage fraction, *k* ∈ (0.906, 1), we will have $R_{c}^{min}$ ∈ ($\sqrt{3}$*R_s_*, 2*R_s_*). Having *R*_c_ ≥ $R_{c}^{min}$ is both a necessary and sufficient condition to ensure that *k-coverage* implies connectivity, according to [10]. From [12], the optimal tessellation, using regular triangles with a side length equal to $\sqrt{3}$*R_s_*, ensures complete area coverage.

In the case of a random deployment strategy (i.e., nodes’ coordinates stem from a Gaussian distribution), the initial AoI coverage is relatively low. In that case, node relocation is imperative to extend the network lifetime and maximize coverage. There is a trade-off between the lifetime extension rate and the coverage rate. The more we respect joint coverage and communication constraints, the better the lifetime we achieve. Since we also want to examine how the network lifetime is affected by a network-scaling factor in the case of large networks, we applied a multihop communication pattern. Under this assumption, the Dijkstra algorithm is used, with the edge weights calculated according to the energy consumption Equations (1) and (2). Overall, we define the following objective function, *F*, which we wish to minimize, as follows:

(9) $$\min F=f_{1}+\frac{1}{f_{2}}\;s.t.:\;P_{ri}\geq P_{rthres}\;\forall i\in \left[1\ldots N\right]$$

### 3.3. Neural Network Framework

**Figure 2:**
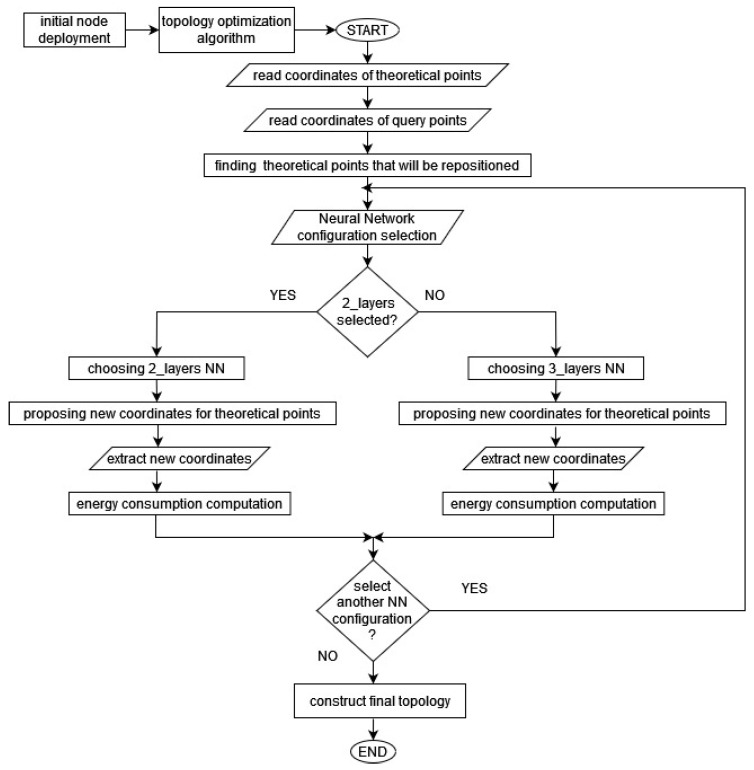
Flowchart diagram of neural network solver.

We solved our combinatorial optimization problem, as defined in (9), by building a neural network framework in Python, which utilizes well-known libraries such as Numpy, TensorFlow, and Keras. In Figure 2, we give a flowchart diagram of the proposed neural network solver. For the initialization phase, we use csv files as input datasets to express the sensors’ initial coordinates. These coordinates, which form the initial topology, are the theoretical topology points and are given as an input to the solver in the first execution step of the algorithm. The algorithm reads a digital map of the FoI and extracts the sensor node coordinates to a csv file. This map also provides the coordinates of the query or reserved points.

The algorithm’s basic operation is to propose the nearest-neighbor theoretical points for every query point. This is achieved with a single-layer perceptron, i.e., a feed forward neural network (FFNN) with many inputs and outputs for each query point. In the case of a deterministic topology, the inputs to the FFNN are the coordinates of query points and the coordinates of theoretical points, i.e., the ones resulting from the CVT. The FFNN’s outputs are the sensor coordinates of the theoretical points which are as close as possible to each query point, with respect to the communication and coverage constraints defined in the previous section. Therefore, the algorithm selects its nearest (i.e., energy-efficient) neighbor for each node.

In our work, two NN configurations were built to propose the new and energy-efficient coordinates for each reserved sensor terrain point. The structure of the first NN configuration consists of (2) layers, and the second NN of (3) layers. We built a deep learning NN model with different parameters in Python using Keras [32]. Keras is a powerful, easy-to-use, free, and open source Python library for developing and evaluating deep learning models. It is part of the TensorFlow library and allows us to define and train NN models. We used the classes Sequential and Dense from Keras in our model. Models in Keras are defined as a sequence of layers. We created a sequential model and added layers. The best network structure is found through a process of trial-and-error experimentation [13].

Compiling a NN model uses the efficient numerical libraries of TensorFlow. The backend automatically chooses the best way to represent the network for training and making predictions. During compilation, we specified some additional properties required for the network training. Training a network means finding the best set of weights to map inputs to outputs for our dataset. We trained or fitted our model on our loaded data by calling the fit() function on the model. After training the model, we used it to make predictions on new data using the method model.predict(). Theoretical points will be repositioned in the new coordinates proposed by the NN.

The criterion for a valid geometric topology is that the proposed relocation of each node should be at a distance of L ≤ (2 − $\sqrt{3}$) × *R_s_* from its original positions and towards the orientation direction of the sink node. The neural network solver proposed a network topology with the minimum energy consumption, according to the objective function in Equation (9). We then extracted the nodes’ coordinates to a csv file and we could draw the new topology. As soon as the solver reached an energy-efficient (i.e., final) topology, we proceeded with the calculation of the total network lifetime, according to the final topology design. Based on the trained NN model, the new nodes’ coordinates indicate points as close as possible to the sink nodes, regarding the communication and coverage constraints. This means that we may relocate some nodes apart from their initial points, as suggested by the CVT. With the proposed algorithm, we managed to increase the network lifetime in all the proposed use cases at a factor of 3% to 5%, ensuring a connectivity rate of over 90% in all topologies. Clearly, there is a trade-off between the lifetime extension and coverage percentage as soon as constraints are fulfilled, and the objective function gets lower for each topology. Therefore, node relocation is a function of that trade-off.

On the contrary, the situation is different for an initial random topology deployment. In that case, nodes’ coordinates are derived, for example, from a Gaussian distribution, and are initially scattered around near the sink node. To this end, the coverage rate is low. The NN solver needs to stretch the initial topology to the extent where the final sensor coordinates fulfil both coverage and connectivity constraints and extend the network lifetime.

**Figure 3:**
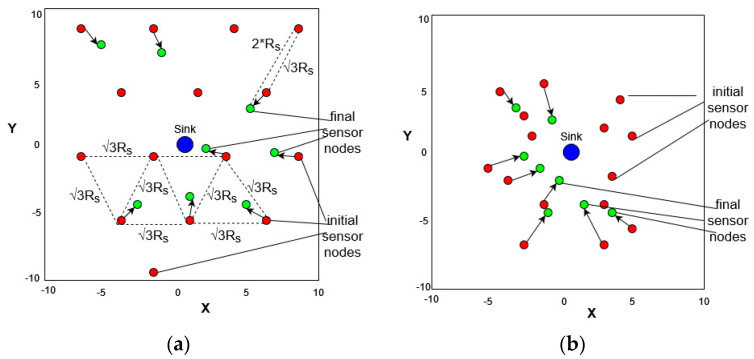
(**a**) Final placement of sensor nodes for a deterministic deployment, sink node at point (0, 0); (**b**) Final placement of sensor nodes for a random deployment, sink node at point (0, 0).

In Figure 3, we depict a snapshot topology example of (a) a deterministic topology and (b) a random topology, in both of which the sink node is placed at (0, 0) in the middle of the FoI. In case (a), we depict the initial node positions after the relocation proposed by the solver, in respect of the constraints, as red dots and the final ones as green dots. We clearly observed that, in this case, the solver needs to evaluate the trade-off between energy consumption and coverage rate, and it turns out that the relocation is close to the initial points, which are the ones proposed by the CVT topology, which is the best one in terms of geometry; in contrast, in case (b), the solver starts from a random topology and has more degrees of freedom to relocate nodes according to the constraints and to extend the network lifetime.

## 4. Use Case Scenarios and Simulations

In this section, we will provide all necessary details and simulation results related to various topologies’ use case scenarios. In particular, we will focus on three distinct cases: (1) a small-size network with size of 20 m × 20 m and consisting of 15 nodes; (2) a medium-size network with a size of 110 m × 110 m and consisting of 428 nodes; and (3) a large-size network with a size of 150 m × 150 m and consisting of 800 nodes. For each use case, the solver estimates the total number of nodes to relocate so that the network lifetime is extended and the rate of that increases. In this work, all use cases and network types focus on application in smart vineyard precision agriculture. This means that the FoI relates to crop arrays in which we need to measure several environmental values, which may affect grape diseases. Moreover, node locations highly depend on these crop arrays, as in [28]. We also give this estimate for a solver consisting of two and three hidden layers, respectively. Furthermore, in Table 1, we give all the necessary simulation parameters, definitions, and values, as used by the NN solver.

### 4.1. Use Case 1: Small-Size Network

A small-size network of 20 × 20 m^2^, with 15 nodes and 1 sink node, was fed to the NN solver, which consisted of two hidden layers (use case 1a). We used a deterministic topology, as proposed by a CVT (i.e., Voronoi) graph. This means that according to the Voronoi graph theory, we placed 15 nodes at the vertexes of hexagons in order to cover an area of 20 × 20 m^2^.

According to Table 2, which gives parameters and values for the simulation, the sink node was placed at (0, 0); for case 1a, we used two hidden layers, and for case 1b, we used three hidden layers. Since we had eight reserved points, the solver relocated 8 nodes out of 15 nodes, which means that almost 53.34% of the topology nodes changed positions, lowering the objective function value. According to the simulation:

1. The initial topology leads to a network lifetime of 8678 rounds.
2. The final topology, as proposed by the solver with (2) hidden layers, leads to a network lifetime of 9039 rounds, which is an increase of 4.16%.
3. The final topology, as proposed by the solver with (3) hidden layers, leads to a network lifetime of 9094 rounds, which is an increase of 4.8%.

**Figure 4:**
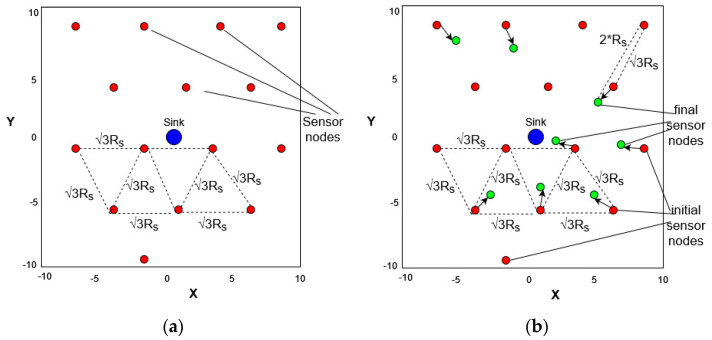
(**a**) Initial placement of sensor nodes for a deterministic deployment, sink node at point (0, 0); (**b**) Final placement of sensor nodes for a deterministic deployment, sink node at point (0, 0).

In Figure 4, we depict a snapshot of the topology for the example of use case 1a. The red dots represent the nodes at their initial positions and the green dots represent the (8) relocated nodes, according to the solver’s decision based on the three hidden layers. The snapshot of the (b) topology leads to a total network lifetime of 9094 rounds and an increase of 4.8%.

### 4.2. Use Case 2: Medium-Size Network

A medium-size network of 110 × 110 m^2^, with 428 nodes and 1 sink node, was fed to the NN solver, which consisted of two hidden layers (use case 1a). We used a deterministic topology, as proposed by a CVT (i.e., Voronoi) graph. This means that according to the Voronoi graph theory, we placed 428 nodes at the vertexes of hexagons in order to cover an area of 110 × 110 m^2^.

According to Table 3, which gives parameters and values for the simulation, the sink node was placed at (0, 0); for case 2a, we used two hidden layers, and for case 2b, we used three hidden layers. Since we had 150 reserved points, the solver relocated 150 nodes out of 428 nodes, which means that almost 35% of the topology nodes changed positions, lowering the objective function value. According to the simulation:

1. The initial topology leads to a network lifetime of 1646 rounds.
2. The final topology, as proposed by the solver with (2) hidden layers, leads to a network lifetime of 1710 rounds, which is an increase of 3.89%.
3. The final topology, as proposed by the solver with (3) hidden layers, leads to a network lifetime of 1716 rounds, which is an increase of 4.25%.

### 4.3. Use Case 3: Large-Size Network

A large-size network of 150 × 150 m^2^, with 800 nodes and 1 sink node, was fed to the NN solver, which consisted of two hidden layers (use case 1a). We used a deterministic topology, as proposed by a CVT (i.e., Voronoi) graph. This means that according to the Voronoi graph theory, we placed 800 nodes at the vertexes of hexagons in order to cover an area of 150 × 150 m^2^.

According to Table 4, which gives parameters and values for the simulation, the sink node was placed at (0, 0); for case 3a, we used two hidden layers, and for case 3b, we used three hidden layers. Since we had 406 reserved points, the solver relocated 406 nodes out of 800 nodes, which means that almost 50.75% of the topology nodes changed positions, lowering the objective function value. According to the simulation:

1. The initial topology leads to a network lifetime of 1181 rounds.
2. The final topology, as proposed by the solver with (2) hidden layers, leads to a network lifetime of 1224 rounds, which is an increase of 3.64%.
3. The final topology, as proposed by the solver with (3) hidden layers, leads to a network lifetime of 1227 rounds, which is an increase of 3.9%.

### 4.4. Use Case 4: Small-Size Network—Random Deployment

A small-size network of 20 × 20 m^2^, with 15 nodes and 1 sink node, was fed to the NN solver, which consisted of two hidden layers (use case 1a). We used a random topology, which means that we placed 15 nodes inside the area of 20 × 20 m^2^, the coordinates of which derive from the Gaussian distribution.

According to Table 5, which gives parameters and values for the simulation, the sink node was placed at (0, 0); for case 4a, we used two hidden layers, and for case 4b, we used three hidden layers. The solver relocated 8 nodes out of 15 nodes, which means that almost 53.34% of the topology nodes changed positions, lowering the objective function value. According to the simulation:

1. The initial topology leads to a network lifetime of 11,896 rounds.
2. The final topology, as proposed by the solver with (2) hidden layers, leads to a network lifetime of 12,443 rounds, which is an increase of 4.6%.
3. The final topology, as proposed by the solver with (3) hidden layers, leads to a network lifetime of 12,505 rounds, which is an increase of 5.2%.

### 4.5. Aggregating the Results

**Figure 5:**
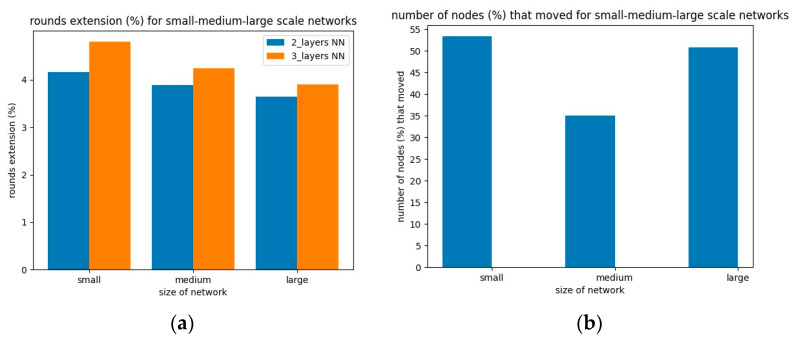
(**a**) Network lifetime extension rate per network size type and use case; (**b**) Total node relocation percentage per network size type.

Aggregating the previous simulation results related to the total network lifetime extension, we plot the extension per network size type for each use case and the solver’s number of hidden layers in Figure 5a. The lifetime extension depicts the comparison, in terms of total energy consumption, of the initial and final topologies per use case. All the initial topologies are based on the deterministic case, i.e., the CVT (Voronoi) graph topology.

According to Figure 5a, the solver managed to extend the network lifetime in all use cases and for all network size types, which means that the algorithm scales polynomially. Moreover, we observed that the choice of three instead of two solver hidden layers led to a better network lifetime extension in all cases. In that sense, for the deterministic network types (i.e., the Voronoi graphs), the addition of one more hidden layer in the solver, in conjunction with the corresponding tuning of the model’s training parameters, led to a longer network lifetime. In Figure 5b, we plot the percentage (%) of the relocated nodes, in each of the network type use cases, as compared to the total nodes of the initial topologies.

For the cases of deterministic CVT initial topologies, no matter how much we tried to increase the number of relocating nodes, we did not actually succeed in further extending the network lifetime. This is due to the fact that the solver also tried to extend the area coverage; therefore, it did not have many degrees of freedom to move nodes to new locations. To this end, we obtained a final topology in polynomial time, but the relocation rate stayed low. Additionally, in dense sensor areas of interest, by relocating 46.38% of the total nodes on average, we extended the network lifetime by an average of 4.5%.

**Figure 6:**
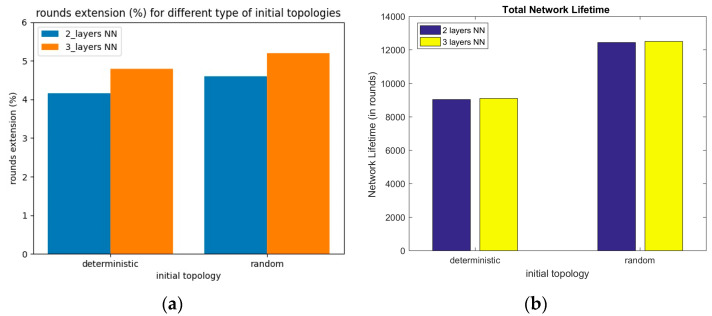
(**a**) Network lifetime extension rate for different network types and initial topologies (i.e., deterministic vs. random), for (2) and (3) hidden solver layers; (**b**) Network lifetime in rounds for deterministic and random topologies.

In Figure 6a, we plot the network lifetime extension rate, comparing 2-layer and 3-layer solvers, for the deterministic and random use case network topologies. Firstly, we can observe that the 3-layer NN configuration led to a greater lifetime extension in both cases. Secondly, for the random topology deployment (i.e., Gaussian), we obtained a better lifetime extension rate in both NN configurations. This is because in random topologies, the solver has more degrees of freedom to relocate nodes than in the deterministic case. This means that the solver outputs a better topology deployment, in terms of objective function, under the constraints. In Figure 6b, we plot the actual lifetime values, in rounds, for these cases. Again, we can observe that for random initial topologies, the solver achieved 12,443 and 12,505 rounds for the 2-layer and 3-layer NN configuration, respectively.

Table 6 presents the values of total energy consumption per round and the network lifetime for the initial and final topologies, respectively, for each use case. We can observe that the solver managed to reduce the total consumed energy; additionally, the choice of three instead of two solver hidden layers led to a greater reduction in all cases. This means that more total residual energy per round was ensured and the network could remain functional for more rounds until all the nodes died.

## 5. Conclusions

In the published literature, there are techniques in WSNs for joint coverage and energy optimization after the initial sensor deployment [33,34,35,36,37]. Having these techniques in mind, in this paper, we proposed a near-optimal solution in polynomial time applying NN configurations for the problem of joint sensor network energy and coverage constraint lifetime optimization. Therefore, we considered and compared several WSN topologies, in conjunction with several NN parameter configurations, and tested them in terms of the total network lifetime extension rate. This means that our solver tried to relocate a percentage of sensor nodes in order to minimize an objective function under certain coverage and connectivity constraints.

Our simulations focused on three distinct and representative use cases, related to small-, medium-, and large-size network deployments, for precision agriculture applications. The simulation results show that our proposed solver achieved a network lifetime extension in all use cases while maintaining the constraints. The network lifetime extension factor was around 5% in all cases, ensuring a coverage percentage of 90% in all topologies. Moreover, in all network cases, the connectivity among the communicating node pairs was ensured. Our simulations show a trade-off between lifetime extension and coverage in terms of optimization constraints. This trade-off is a system parameter, which the network designer needs to fine-tune. A greater extension was achieved in the cases where the solver applied more hidden layers. In future work, we will consider experimenting with more NN configurations and parameter settings and enhance our communication model with power control optimization in some of the cases. A limitation of our proposed solution is that we tested it in a simulation environment. However, the solver was responsive to large WSN deployments. In essence, our work proposes a tool for a potential network designer to predefine and, afterwards, deploy the final topology.

In future work, we plan to test our solution in real field conditions and discuss the potential limitations associated with the utilization of moving sensors for the final placement, under hybrid topologies with static nodes and moving ones. Moreover, we plan to test the solver’s robustness, as far as several neural network configurations are concerned, and highlight which is the best for each network scenario.

## Figures and Tables

**Table 1:** Objective functions and solver’s simulation parameters.

Parameters	Definitions	Values
Sensing radius	Sensing radius of sensor nodes	3 m
pk	Packet size (in bits)	1500
initial_energy	Initial energy at every node’s battery	2
dataset	Data file with coordinates of theoretical points	coordinates.csv
query_points	Data file with coordinates of query points	query_points.csv
e_elec = e_s_ = e_t_ = e_r_	Energy dissipated at the electronic transceiver (/bit)	50 nJ/bit
β	Transmission quality estimation	0.1 nJ/bit/m^2^
α	Signal degrading factor 2 ≤ α ≤ 5	3

**Table 2:** Simulation parameters for use case 1: small-size network.

Parameters	Values
Type of topology	Deterministic
Deployment area	20 m × 20 m
Sensing area width	20
Sensing area length	20
Topology type	CVT
Number of initial nodes	15
Number of relocated nodes	8
Sink node coordinates	(0, 0)
Solver’s hidden layers	2 (case 1a) and 3 (case 1b)

**Table 3:** Simulation parameters for use case 2: medium-size network.

Parameters	Values
Type of topology	Deterministic
Deployment area	110 m × 110 m
Sensing area width	110
Sensing area length	110
Topology type	CVT
Number of initial nodes	428
Number of relocated nodes	150
Sink node coordinates	(0, 0)
Solver’s hidden layers	2 (case 2a) and 3 (case 2b)

**Table 4:** Simulation parameters for use case 3: large-size network.

Parameters	Values
Type of topology	Deterministic
Deployment area	150 m × 150 m
Sensing area width	150
Sensing area length	150
Topology type	CVT
Number of initial nodes	800
Number of relocated nodes	406
Sink node coordinates	(0, 0)
Solver’s hidden layers	2 (case 3a) and 3 (case 3b)

**Table 5:** Simulation parameters for use case 4: small-size network—random deployment.

Parameters	Values
Type of topology	Random
Deployment area	20 m × 20 m
Sensing area width	20
Sensing area length	20
Number of initial nodes	15
Number of relocated nodes	8
Sink node coordinates	(0, 0)
Solver’s hidden layers	2 (case 4a) and 3 (case 4b)

**Table 6:** Total energy consumption and network lifetime values for every use case.

Use Case	Initial Energy at Every Node’s Battery (J)	Number of Initial Nodes	Network Lifetime (Rounds)	Total Energy Consumption (mJ/Round)
Use case 1	2	15	8678	3457
Use case 1a (two hidden layers)	2	15	9039	3319
Use case 1b (three hidden layers)	2	15	9094	3299
Use case 2	2	428	1646	520
Use case 2a (two hidden layers)	2	428	1710	500
Use case 2b (three hidden layers)	2	428	1716	498
Use case 3	2	800	1181	1354
Use case 3a (two hidden layers)	2	800	1224	1307
Use case 3b (three hidden layers)	2	800	1227	1304
Use case 4	2	15	11,896	2522
Use case 4a (two hidden layers)	2	15	12,443	2411
Use case 4b (three hidden layers)	2	15	12,505	2399
